# The effects of childhood trauma on personality in unaffected first-degree relatives of patients with major depressive disorder

**DOI:** 10.1186/s12888-022-03909-z

**Published:** 2022-05-03

**Authors:** Yu-jie Wen, Qi-jing Bo, Wen-peng Hou, Zhen Mao, Feng Li, Fan He, Fang Dong, Xin Ma, Yi-lang Tang, Xian-bin Li, Chuan-yue Wang

**Affiliations:** 1 The National Clinical Research Center for Mental Disorders & Beijing Key Laboratory of Mental Disorders, Beijing Anding Hospital, Capital Medical University, No.5 Ankang Lane, Dewai Avenue, Xicheng District, Beijing, 100088 PR China; 2 Advanced Innovation Center for Human Brain Protection, Capital Medical University, Beijing, China; 3 Mental Health Service Line, Atlanta VA Medical Center, Decatur, GA 30033 USA; 4 Department of Psychiatry and Behavioral Sciences, Emory University, Atlanta, GA USA

**Keywords:** Childhood trauma, Personality, Major depressive disorder, First-degree relatives

## Abstract

**Objectives:**

This study aimed to preliminarily and exploratorily examine the associations between childhood trauma (CT), its subtypes, and personality traits among unaffected first-degree relatives (FDR, children, or siblings) of patients with major depressive disorder (MDD).

**Methods:**

The study sample included three subgroups: MDD patients (*N* = 85), Patients’ FDRs (*N* = 35), and healthy control individuals (HC, *N* = 89). The Childhood Trauma Questionnaire (CTQ) was used to assess childhood trauma and the Eysenck Personality Questionnaire was used to assess personality traits.

**Results:**

Significant differences were found in a few personality traits (*p* < 0.05 for extraversion, neuroticism, and psychoticism) among MDD patients, FDR, and HC, and there were no significant differences between HC and FDR. In the FDR group, compared with those without CT, participants with CT scored significantly higher for neuroticism (N) (*F* = 3.246, *p* = 0.046). CT was significantly associated with N, psychoticism (P) and Lie (L), and the strongest association was between CT total score and N. Significantly positive correlations were found between N and sexual abuse (SA) (*r* = 0.344, *p* = 0.043), emotional neglect (EN) (*r* = 0.394, *p* = 0.019), physical neglect (PN) (*r* = 0.393, *p* = 0.019), and CTQ total score (*r* = 0.452, *p* = 0.006); between P and CTQ total score (*r* = 0.336, *p* = 0.049); and significant negative correlations were found between L and EN (*r* = -0.446, *p* = 0.007), CTQ total score (*r* = -0.375, *p* = 0.027).

**Conclusion:**

In unaffected FDRs, there were significant associations between childhood trauma and a few personality traits, including neuroticism, psychoticism, and lie, and emotional neglect was significantly associated with neuroticism.

**Supplementary Information:**

The online version contains supplementary material available at 10.1186/s12888-022-03909-z.

## Significant outcomes

In first-degree relatives (FDRs) of patients with major depressive disorder (MDD), childhood trauma (CT) was associated with a higher score on neuroticism, psychoticism, and lie, with the association with neuroticism being the strongest. The subtype of emotional neglect had the strongest association with neuroticism.

## Limitations

(1) Data were collected using a cross-sectional questionnaire without independent authentication. (2) The sample size was relatively small, therefore the findings should be considered to be preliminary and exploratory.

## Introduction

Numerous studies have supported the link between personality and the presence of major depressive disorder (MDD) [1, 2]. Compared with non-affected controls, patients with MDD often have higher scores on neuroticism [3], and the score tends to decrease when patients show improvement or achieve remission [4]. Besides, a higher score on neuroticism has been found to be predictive of poorer outcomes in patients with MDD [5–7]. Population-based twin studies showed that the association between neuroticism and MDD may be in part due to shared genetic factors, and they found the genetic correlation with neuroticism was 0.46–0.47 based on their study of 20,692 same-sex twin pairs in Sweden [8]. Personality traits appear to be associated with the onset and course of MDD [9, 10]. However, there have been scarce data on the effects of childhood trauma on personality in first-degree relatives (FDRs) of patients with MDD, and the findings of the few studies are inconsistent [11, 12].

Studies have found personality traits, especially maladaptive traits of adults are associated with childhood trauma (CT) in the general population. De Carvalho et al. found that emotional neglect was associated with reduced reward dependence and persistence [13]. Another study found that emotional abuse was most pervasively related to personality, and neuroticism was significantly associated with emotional abuse and neglect, physical abuse and neglect, and sexual abuse [14]. Emotional abuse was associated with neuroticism in men more profoundly than in women [14]. Our previous study in a sample of adolescents found that there were significant positive correlations between neuroticism score on the Eysenck Personality Questionnaire (EPQ) and CTQ-SF total score, as well as the subscale scores of emotional abuse, and sexual abuse [15].

Many studies have reported that childhood trauma (CT) is a risk factor for MDD. A meta-analysis of prospective cohort studies found that CT was significantly associated with the risk of depression in adults [16]. CT has also been found to be associated with the severity in patients with MDD [17]. Moreover, CT has also been found to be a poorer clinical course, earlier age of onset [18, 19], episode persistence, and recurrence [20] in patients with MDD. Recent studies found that personality may mediate the effects of childhood abuse on the severity of depressive symptoms in patients with MDD [21, 22] as well as the general population [23–26]. Personality traits such as neuroticism, extraversion, hopelessness, and external locus of control have been reported to mediate the relationship between CT and 4-year remission of depressive and anxiety disorders [27].

These studies all suggest that there are complex associations between CT, personality, the severity of depressive symptoms in patients with MDD. However, few studies have examined the associations between CT and personality traits in FDRs of patients with MDD. Therefore, this study was designed to address this gap. The Childhood Trauma Questionnaire-Short Form (CTQ-SF) was used to assess the characteristics of childhood trauma and the EPQ was used to assess personality traits. Our hypotheses are: (1) FDRs have more prominent maladaptive personality traits compared with HC, and CT and/or its subtypes are associated with different personality traits in FDRs, (2) CT is associated differently with personality traits in patients with MDD, HC, and FDRs.

## Methods

### Participants

This was a cross-sectional study conducted at Beijing Anding Hospital, Capital Medical University, Beijing, China. The protocol was reviewed and approved by the ethics committee of Beijing Anding Hospital, Capital Medical University. All participants provided their informed consent to participate in the study after being informed about the purpose of the study.

All participants were recruited between September 2014 and September 2016. The study sample consisted of three subgroups: patients with MDD (*n* = 85); FDRs of MDD patients (*n* = 35); and unaffected healthy control (HC, *n* = 89). The Structured Clinical Interview for DSM-IV Axis I disorders-Patient Edition (SCID-I/P) was used for diagnosis, and the diagnosis of MDD was made according to the Diagnostic and Statistical Manual of Mental Disorders, Fourth Edition (DSM-IV) [28]. The FDR and HC recruited had no current Axis I diagnosis of psychiatric or cognitive disorders.

The inclusion criteria for the study were: (1) aged between 16 and 55; (2) received more than 9 years of formal education and was able to understand and willing to sign an informed consent. Participants with any of the following were excluded: (1) with a current diagnosis of substance abuse or dependence (not including nicotine); (2) with an unstable, major medical or neurological condition; (3) had received electroconvulsive therapy within the past three months.

Based on the results of Childhood Trauma Questionnaire-Short Form (CTQ-SF): The CT positive subgroup consisted of individuals who had one or more subscores reaching moderate or severe levels of trauma, all participants in three groups (HC, FDR, and MDD) were divided into 2 independent subgroups (CT positive subgroup and CT negative subgroup). The CT negative subgroup consisted of individuals who either scored in the low or no category on the CTQ-SF.

### Instruments

#### Childhood Trauma Questionnaire-Short Form (CTQ-SF)

The CTQ-SF is a 28-item self-report retrospective inventory intended to measure abuse and neglect of children ages 12 and older [29, 30]. The Chinese version used has been tested and shown with good reliability and validity in the general population [31, 32]. It is a 5-point Likert scale ranging from “Never true” to “Very often true”. The CTQ-SF contains five subscales, which measure three types of abuse and two types of neglect: namely emotional abuse (EA), physical abuse (PA), sexual abuse (SA), emotional neglect (EN), and physical neglect (PN). The score for each scale is a sum of scores of specific items, and the total score of the CTQ-SF is a sum of scores on all scales. The severity of each trauma category based on cut-off scores was quantified as “none (or minimal)”, “low (to moderate)”, “moderate (to severe)” and “severe (to extreme)”. In this study, cut-off scores for “moderate (to severe)” were employed to classify study participants as positive for a history of specific trauma category. The CTQ cut-off scores for “moderate (to severe)” are as follows: EA ≥ 13, PA ≥ 10, SA ≥ 8, EN ≥ 15, and PN ≥ 10.

#### Eysenck Personality Questionnaire (EPQ)

The EPQ is a self-report questionnaire for measuring personality dimensions (traits), including a junior version (for 7–15 years old) and an adult version (for 16 years and older) [33]. The validated Chinese version of EPQ for adults has 88 questions and it has been tested to show good reliability and validity [34]. The EPQ consists of 4 personality dimensions (traits): (1) extraversion/introversion (E), with a higher score meaning greater extroversion; (2) neuroticism/stability (N), with a higher score meaning less stable emotions; (3) psychoticism/socialization (P), with a higher score meaning higher psychoticism; (4) lie/social desirability (L), with a higher score meaning higher tendency for dissimulation and fake on the responses. The score for each dimension is a sum of the responses (“*agreement*” scored 1, “*disagreement*” scored 0) to specific questions.

#### Health Questionnaire (PHQ-9)

Health Questionnaire (PHQ-9), a nine-item instrument, was purposed primarily for the application in primary care [35]. It was developed by referencing the diagnostic standard applied for the assessment of depression-induced disorder, as cited from the 4th edition of the Diagnostic and Statistical Manual [36]. The involved items are subjected to ratings ranging from not at all to almost every day for the most recent two weeks with a four-point scale for the duration. The PHQ-9 has been used widely for screening, diagnosis, monitoring treatment response.

### Statistical analysis

Statistical analyses were conducted using the SPSS (version 19.0). All differences were considered statistically significant when *p* < 0.05 for both directions. The continuity-adjusted χ^2^ test and the Fisher’s exact test were used to compare the distribution of categorical variables among the three groups and between groups with and without CT. The Kruskal–Wallis test was performed to compare the age and education level of the three groups. The t-test and the Mann–Whitney test were performed to compare age, education level, E, N, P, L scores between three groups with and without CT. Pearson’s correlation and spearman’s correlation (for non-normally distributed data) were adopted to assess the association between personality traits and types of CT in FDR and the other two groups.

## Results

### Sociodemographic characteristics

There were no significant differences in sex, age and education level between the three groups (Table 1). There was a significant difference in age (*P* = 0.02) between those with and without CT in FDRs (based on the cut-off), but no significant differences in other aspects (Table 2).

**Table 1:** Sociodemographic characteristics of the three groups

**Characteristic**	**HC (*n* = 89)**	**FDR (*n* = 35)**	**MDD (*n* = 85)**	***P***
Gender (M,%)	37(41.57%)	15(42.86%)	39(45.88%)	0.845
Age (Y; mean, SD)	33.72(10.28)	33.20(9.21)	33.36(10.73)	0.959
Education level (Y; mean, SD)	14.27(3.02)	13.74(2.94)	13.44(3.11)	0.194
PHQ^1^	3^a^	3^b^	10^c^	< 0.001^#^, a, b < c
CTQ-SF(mean, SD)	34.56(6.92)^a^	35.89(7.47)^b^	41.72(11.79)^c^	< 0.001^#^, a, b < c
EA	6.06(1.59)^a^	5.54(1.15)^b^	7.95(3.72)^c^	< 0.001^#^, a, b < c
PA	5.60(1.41)^a^	5.26(0.98)^b^	6.29(2.71)^c^	0.014^*^, a, b < c
SA	5.38(0.96)	5.71(1.64)	5.88(2.66)	0.227
EN	9.58(3.49)^a^	10.43(3.74)^b^	12.31(4.78)^c^	< 0.001^#^, a, b < c
PN	7.93(3.26)^a^	8.94(3.00)^b^	9.28(3.12)^c^	0.017^*^, a < c
EPQ(mean, SD)				
E	57.77(9.43)^a^	55.43(11.75)^b^	45.38(9.80)^c^	< 0.001^#^, a, b > c
N	41.50(11.22)^a^	40.68(8.43)^b^	55.66(11.71)^c^	< 0.001^#^, a, b < c
P	46.64(9.71)^a^	45.64(13.16)^b^	50.49(9.38)^c^	0.016^*^, a, b < c
L	47.20(9.59)	47.23(11.84)	45.60(9.58)	0.520

Data are shown as a percentage or mean and standard deviation (SD)

*HC* healthy controls, *FDR* first-degree relatives of patients with major depressive disorder, *MDD* patients with major depressive disorder, *M* male, *%*, percentage, *Y* years, *PHQ* Patient Health Questionnaire, *CTQ-SF* Childhood Trauma Questionnaire short form, *EPQ* Eysenck Personality Questionnaire, *E* extraversion, *N* neuroticism, *P* psychoticism, *L* lie

^1^Kruskal-Wallis Tests, ^a^healthy controls group, ^b^first-degree relatives of patients with major depressive disorder group, ^c^patients with major depressive disorder group

^*^ *P* < 0.05, #*P *< 0.01. After Bonferroni correction

**Table 2:** Sociodemographic characteristics in First-degree relatives with and without CT

Characteristic	With CT (*n* = 15)	Without CT (*n* = 20)	χ^2^/t/u/F	*P Cohen’s d*
Gender (M, %)	6(40.00%)	9(45.00%)	0.087	0.767 /
Age (Y; mean, SD)	37.33(8.72)	30.10(8.50)^*^	2.464	0.019 -0.841
Education level (Y; mean, SD)	12.87(2.88)	14.40(2.89)	-1.556	0.129 0.53
PHQ^a^	3	2	102.5	0.114 /
EPQ (mean, SD)				
E	57.09(9.62)	54.18(13.23)	0.718	0.478 -0.246
N^b^	44.43(7.89)	37.87(7.86)	3.246	0.046^*^ -0.833
P	48.43(18.41)	43.55(7.01)	1.091	0.283 -0.372
L	46.03(13.24)	48.13(10.95)	-0.512	0.612 0.175

Data are shown as a percentage or mean and standard deviation (SD)

*HC* healthy controls, *FDR* first-degree relatives of patients with major depressive disorder, *MDD* patients with major depressive disorder, *M* male, *%* percentage, *Y* years, *PHQ* Patient Health Questionnaire, *EPQ* Eysenck Personality Questionnaire, *E* extraversion, *N* neuroticism, *P* psychoticism, *L* lie

^a^Mann-Whitney U tests, ^b^Covariance analysis * *P* < 0.05, #*P* < 0.01

### Personality dimensions in individuals with and without CT

There were no significant differences in personality traits between FDRs and HC. Compared with FDR and HC, patients with MDD displayed significantly higher scores on E, N, and P (*P* < 0.001 for E, N; *P* = 0.016 for P). Higher severity of CT was also found in the MDD group than that in FDR and HC (*P* < 0.001), as well as a higher severity in 4 subtypes of CT: EA, PA, EN, and PN (*P* < 0.001 for EA, PA, and EN; *P* = 0.017 for PN). Details please see Table 1.

People with CT had significantly higher N scores than those without CT in FDRs (*F* = 3.246, *p* = 0.046, Cohen’s d = -0.833) (Table 2). We searched for outliers and conducted a sensitivity analysis for the results in Table 2. Compared with those without CT (*n* = 18), participants with CT (*n* = 13) scored significantly higher for Neuroticism (n) (*F* = 0.368, *P* = 0.046), suggesting the findings were stable.

### Association between CT subtypes and personality dimensions

In FDR, significantly positive correlations were found between N and SA (*r* = 0.344, *p* = 0.043), N and EN (*r* = 0.394, *p* = 0.019), E and PN (*r* = 0.393, *p* = 0.019), and E and CTQ total score (*r* = 0.452, *p* = 0.006), and between P and CTQ (*r* = 0.336, *p* = 0.049). Significantly negative correlations were found between L and EN (*r* = -0.446, *p* = 0.007), L and CTQ (*r* = -0.375, *p* = 0.027) (Table 3).

**Table 3:** Association between childhood maltreatment types and personality dimensions in FDR

EPQ	EA	PA	SA	EN	PN	CTQ-SF
E	-.030	.220	.072	-.030	.032	.068
N	.159	.142	.344^*^	.394^*^	.393^*^	.452^#^
P	.224	.159	.189	.231	.292	.336^*^
L	-.023	-.075	-.160	-.446^#^	-.275	-.375^*^

*EPQ* Eysenck Personality Questionnaire, *EA* emotional abuse, *PA* physical abuse, *SA* sexual abuse, *EN* emotional neglect, *PN* physical neglect, *CTQ-SF* Childhood Trauma Questionnaire short form, *E* extraversion, *N* neuroticism, *P* psychoticism, *L* lie

^*^P < 0.05, #*P* < 0.01. Pearson’s correlation and Spearman’s correlation were performed

In HC, significantly positive correlations were found between N and SA (*r* = 0.368, *p* = 0.002), as well as CTQ (*r* = 0.290, *p* < 0.001); and between P and EA (*r* = 0.293, *p* = 0.011), P and PN (*r* = 0.303, *p* = 0.005), and P and CTQ (*r* = 0.292, *p* = 0.002). Significant negative correlations were found between L and EA (*r* = -0.256, *p* = 0.015), L and SA (*r* = -0.258, *p* = 0.015), L and EN (*r* = -0.282, *p* = 0.008), L and PN (*r* = -0.248, *p* = 0.019), and L and CTQ total score (*r* = -0.286, *p* = 0.007).

In patients with MDD, positive correlations were found between N and EA (*r* = 0.333, *p* = 0.002), N and PA (*r* = 0.275, *p* = 0.011), N and CTQ total score (*r* = 0.223, *p* = 0.041); P and EA(*r* = 0.309, *p* = 0.004), P and SA (*r* = 0.356, *p* = 0.001), P and CTQ total score (*r* = 0.240, *p* = 0.027). Significant negative correlations were found between E and EA (*r* = -0.397, *p* < 0.001), E and EN (*r* = -0.325, *p* = 0.007), E and PN (*r* = -0.246, *p* = 0.023), E and CTQ total score (*r* = -0.388, *p* < 0.001); L and EA (*r* = -0.266, *p* = 0.014), L and CTQ total score (*r* = -0.245, *p* = 0.024) (Appendix 1).

We also searched for outliers and conducted sensitivity analysis for the results in Table 3 (Appendix 2). Sensitivity analysis suggested that the results were stable.

## Discussions

This study was the first to preliminarily and exploratorily verify the association between CT and personality traits in unaffected FDRs of patients with MDD. We found no significant differences between FDR and HC in personality traits. In FDRs, CT was associated with a higher score on neuroticism, psychoticism, and lie, with the association with neuroticism the strongest. Besides, in terms of subtype, emotional neglect was the one to be found to have the strongest association with neuroticism.

Many previous studies have demonstrated that a high proportion of patients with severe depression have maladaptive personality traits. Studies have also shown that personality disorders at baseline in patients with MDD were robust predictors of a slow remission [37], and even after they achieved remission, personality disorders were a strong predictor of prospectively of accelerated relapse [37].

However, there have been few studies on personality traits in first-class relatives of patients with MDD and the effects of child abuse and its subtypes on personality traits. In contrast to some prior studies [38, 39], we found no significant differences in the EPQ scores between FDRs and healthy controls. Findings from previous twin studies on personality are consistent in attributing approximately half of the variance in personality to genetic effects, with the remaining variance attributed to environmental factors, with the possibility of gene-environment interactions in personality development [40]. Some studies [11, 39] suggested that, for personality traits, siblings may be no more similar than strangers. Others suggested that genetic and personality factors might be two relatively separate risk factors in the development of depression [40].

Our findings are consistent with Wu et al. [12], which found no familial aggregation in personality traits between 92 patients with MDD and their 190 FDRs. As suggested by Coid [41], a definitive conclusion about the impact of heredity (genetics) and environmental factors on personality has not yet been drawn in recent studies. Some researchers suggested that there was no close association between genetics and personality [42].

We also found no significant differences in childhood trauma between FDRs and the healthy control group. Previous studies have repeatedly supported the association between CT and the risk of developing depression [43, 44]. Our findings may suggest that CT might involve a separate mechanism from genetic factors in depression.

Furthermore, we found that among the FDRs, neuroticism, psychoticism and lie were significantly higher in the group with CT than those without CT, with the difference in neuroticism being most significant. In addition, CT was associated with all the four personality dimensions in MDD patients. To our best knowledge, this is the first study to show significant differences between childhood trauma and personality dimensions in unaffected FDRs. Although we performed outlier and sensitivity analyses to prove the stability of above conclusions, our correlation results were preliminary and exploratory due to the small sample size, and the conclusion “maybe” rather than “definitely” were as described above. Previous studies have shown that personality traits, especially neuroticism, may mediate the effects of CT on MDD [44].

Individuals with CT displayed significantly higher N scores than those without CT in FDRs. As reported by the prior studies [45, 46], CT was associated with higher N scores in both the general population and in patients with MDD. Our findings in the FDRs of patients with MDD add to the current literature. Similar to our previous study [15], the current study showed that personality traits were significantly associated with childhood trauma in FDRs. E, N, P, and L were primarily associated with EN among CT. The subtype of childhood trauma that was most associated with neuroticism was emotional neglect. Different from our findings, a few other studies reported emotional abuse was the one with the strongest association with neuroticism in healthy controls; and in individuals with avoidant personality disorder [43, 44]. Of note, they also found that emotional neglect was associated with neuroticism, secondary to emotional abuse [43, 44]. Additionally, the associations between neglect and multiple personality dimensions have been reported in several prior studies in healthy controls [13]. The differences in different studies may be due to different sample characteristics and different independent variables included in their analysis.

Despite the absence of a full explanation of the neurobiological mechanism of changes to personality in response to CT, some potential mechanisms have been proposed. Individuals who sustained trauma in childhood tend to show a lower level of glucocorticoid expression, in addition to a range of distinct characteristics including changed methylation status in the neuron-specific glucocorticoid receptor promoter, long-lasting hypothalamic–pituitary–adrenal axis change, and excessively active autonomic nervous system [47–49]. As indicated by the different cortisol levels among patients with trauma-induced personality disorder, the processing was considered a potential cause to trigger the distinct coping mechanisms [50, 51].

### Limitations

Several limitations of this study should be acknowledged. First, the data on CT were obtained using the Childhood Trauma Questionnaire-Short Form (CTQ-SF), which is a retrospective self-report questionnaire without independent authentication. Accordingly, the validity of reports might be affected by possible recall biases. Second, our sample size, especially one of the first-degree relatives was relatively small, and this may limit the validity of our findings. Therefore, our findings should be considered to be preliminary and exploratory, and replication studies involving larger samples are needed in the future. Last but not least, this study does not consider how causal pathways could go either one way (traits driving differential susceptibility to the environment) or the other (trauma driving an effect on levels of traits), and further research is needed.

## Conclusions

Childhood trauma is associated with neuroticism, psychoticism, and lie, and emotional neglect is significantly associated with neuroticism in first-degree relatives of patients with MDD. The effects of the personality traits in first-degree relatives of patients with MDD on mental health should also be studied further, and replication studies in larger samples are needed.

## Supplementary Information

**Additional file 1.**

## Data Availability

All data generated or analyzed during this study are included in this article. The data sets used and/or analyzed during the current study are available from the corresponding author on reasonable request.
