# Supplementary material for: The effects of childhood trauma on personality in unaffected first-degree relatives of patients with major depressive disorder
Source: BMC Psychiatry. 2022 May 3;22:312. doi: 10.1186/s12888-022-03909-z (PMC9063055; doi:10.1186/s12888-022-03909-z)
Supplement: Supplementary file 1 — Additional file 1. [file 12888_2022_3909_MOESM1_ESM.docx]

**Appendix 1.** Association between childhood trauma subtypes and personality dimensions in healthy controls and major depression disorder.

1. Association between childhood trauma types and personality dimensions in healthy controls

| **EPQ** | **EA** | **PA** | **SA** | **EN** | **PN** | **CTQ-SF** |
| --- | --- | --- | --- | --- | --- | --- |
| **E** | -0.054 | 0.036 | 0 | -0.188 | -0.168 | -0.147 |
| **N** | 0.202 | 0.206 | .368^#^ | 0.114 | 0.178 | .290^#^ |
| **P** | .293^#^ | 0.052 | 0.196 | 0.094 | .303^#^ | .292^#^ |
| **L** | -.256^*^ | 0.045 | -.258^*^ | -.282^#^ | -.248^*^ | -.286^#^ |

1. Association between childhood trauma types and personality dimensions in patients with major depressive disorder.

| **EPQ** | **EA** | **PA** | **SA** | **EN** | **PN** | **CTQ-SF** |
| --- | --- | --- | --- | --- | --- | --- |
| **E** | -.397^#^ | -.194 | -.094 | -.325^#^ | -.246^*^ | -.388^#^ |
| **N** | .333^#^ | .275^*^ | .102 | .013 | .098 | .223^*^ |
| **P** | .309^#^ | .059 | .356^#^ | .088 | .050 | .240^*^ |
| **L** | -.266^*^ | -.136 | -.209 | -.137 | -.103 | -.245^*^ |

*Note*. EPQ, Eysenck Personality Questionnaire; EA, emotional abuse; PA, physical abuse; SA, sexual abuse; EN, emotional neglect; PN, physical neglect; CTQ-SF, Childhood Trauma Questionnaire short form; E, extraversion; N, neuroticism; P, psychoticism; L, lie.

* P<0.05, #P<0.01. Pearson's correlation correlation were performed.

**Appendix 2.** Association between childhood trauma types and personality dimensions in FDR for sensitivity analysis.

| **EPQ** | **EA** | **PA** | **SA** | **EN** | **PN** | **CTQ-SF** |
| --- | --- | --- | --- | --- | --- | --- |
| **E** | -.144 | .220 | .141 | -.132 | .032 | .149 |
| **N** | .142 | .142 | .312^*^ | .449^#^ | .393^*^ | .452^#^ |
| **P** | .195 | .159 | .160 | .148 | .292 | .336^*^ |
| **L** | -.046 | -.075 | -.044 | -.236 | -.275 | -.375^*^ |

*Note*. EPQ, Eysenck Personality Questionnaire; EA, emotional abuse; PA, physical abuse; SA, sexual abuse; EN, emotional neglect; PN, physical neglect; CTQ-SF, Childhood Trauma Questionnaire short form; E, extraversion; N, neuroticism; P, psychoticism; L, lie.

* P<0.05, #P<0.01. Pearson's correlation and Spearman's correlation were performed.

**Appendix 2.** Multiple linear regression analyses of abuse and neglect scores on the EPQ subscale in healthy controls and major depression disorder.

1. Multiple linear regression analyses of abuse and neglect scores on the EPQ subscale in healthy controls.

| **EPQ subscale** | | ***B*** | ***S. E*** | **Standardized coefficients Beta** | ***t*** | ***P*** |
| --- | --- | --- | --- | --- | --- | --- |
| **N** |  | | | | | |
|  | **Constant** | 11.798 | 7.015 |  | 1.682 | .096 |
|  | **EA** | 2.011 | .698 | .284 | 2.880 | .005 |
|  | **SA** | 3.252 | 1.155 | .278 | 2.817 | .006 |
| **P** |  | | | | | |
|  | **Constant** | 31.871 | 4.262 |  | 7.477 | .000 |
|  | **PN** | .787 | .301 | .264 | 2.614 | .011 |
|  | **EA** | 1.405 | .618 | .230 | 2.272 | .026 |
| **L** |  | | | | | |
|  | **Constant** | 59.543 | 3.826 |  | 15.562 | .000 |
|  | **EA** | -2.034 | .610 | -.336 | -3.333 | .001 |

1. Multiple linear regression analyses of abuse and neglect scores on the EPQ subscale in patients with major depressive disorder.

| **EPQ subscale** | | ***B*** | ***S. E*** | **Standardized coefficients Beta** | ***t*** | ***P*** |
| --- | --- | --- | --- | --- | --- | --- |
| **E** |  | | | | | |
|  | **Constant** | 53.700 | 2.326 |  | 23.087 | .000 |
|  | **EA** | -1.046 | .265 | -.397 | -3.943 | .000 |
| **N** |  | | | | | |
|  | **Constant** | 51.638 | 3.388 |  | 15.240 | .000 |
|  | **EA** | 1.566 | .393 | .498 | 3.985 | .000 |
|  | **EN** | -.685 | .306 | -.280 | -2.240 | .028 |
| **P** |  | | | | | |
|  | **Constant** | 38.321 | 2.852 |  | 13.437 | .000 |
|  | **SA** | 1.144 | .351 | .324 | 3.259 | .002 |
|  | **EA** | .684 | .251 | .271 | 2.728 | .008 |
| **L** |  | | | | | |
|  | **Constant** | 51.039 | 2.388 |  | 21.374 | .000 |
|  | **EA** | -.684 | .272 | -.266 | -2.514 | .014 |

*Note*. EPQ, Eysenck Personality Questionnaire; EA, emotional abuse; PA, physical abuse; SA, sexual abuse; EN, emotional neglect; PN, physical neglect; CTQ-SF, Childhood Trauma Questionnaire short form; E, extraversion; N, neuroticism; P, psychoticism; L, lie. All input independent variables: EA, PA, SA, EN, PN; All input dependent variables: E(extraversion), N, P, L.E(extraversion) is not listed because no independent variables were included.

**A**.N: *R^2^* = .184, Adjusted *R^2^* = .165, *F* = 9.683, *P*<.001. P: *R^2^* = .140, Adjusted *R^2^* = .120, *F* = 6.979, *P*=.002. L: *R^2^* = .113, Adjusted *R^2^* = .103, *F* = 11.109, *P*=.001; **B**. E: *R^2^* = .158, Adjusted *R^2^* = .148, *F* = 15.549, *P*<.001.N: *R^2^* = .162, Adjusted *R^2^* = .142, *F* = 7.950, *P*=.001.P: *R^2^* = .199, Adjusted *R^2^* = .180, *F* = 10.201, *P*<.001.L: *R^2^* = .071, Adjusted *R^2^* = .060, *F* = 6.319, *P*=.014.
